# Levodopa-induced abnormal involuntary movements correlate with altered permeability of the blood-brain-barrier in the basal ganglia

**DOI:** 10.1038/s41598-017-16228-1

**Published:** 2017-11-22

**Authors:** Renata P. Lerner, Veronica Francardo, Koji Fujita, Zisis Bimpisidis, Vincent A. Jourdain, Chris C. Tang, Stephen L. Dewey, Thomas Chaly, M. Angela Cenci, David Eidelberg

**Affiliations:** 1 Center for Neurosciences, The Feinstein Institute for Medical Research, Manhasset, NY 11030 USA; 2 Basal Ganglia Pathophysiology Unit, Department of Experimental Medical Science, Lund University, Lund, Sweden

## Abstract

Chronic levodopa treatment leads to the appearance of dyskinesia in the majority of Parkinson’s disease patients. Neurovascular dysregulation in putaminal and pallidal regions is thought to be an underlying feature of this complication of treatment. We used microPET to study unilaterally lesioned 6-hydroxydopamine rats that developed levodopa-induced abnormal involuntary movements (AIMs) after three weeks of drug treatment. Animals were scanned with [^15^O]-labeled water and [^18^F]-fluorodeoxyglucose, to map regional cerebral blood flow and glucose metabolism, and with [^11^C]-isoaminobutyric acid (AIB), to assess blood-brain-barrier (BBB) permeability, following separate injections of levodopa or saline. Multitracer scan data were acquired in each animal before initiating levodopa treatment, and again following the period of daily drug administration. Significant dissociation of vasomotor and metabolic levodopa responses was seen in the striatum/globus pallidus (GP) of the lesioned hemisphere. These changes were accompanied by nearby increases in [^11^C]-AIB uptake in the ipsilateral GP, which correlated with AIMs scores. Histopathological analysis revealed high levels of microvascular nestin immunoreactivity in the same region. The findings demonstrate that regional flow-metabolism dissociation and increased BBB permeability are simultaneously induced by levodopa within areas of active microvascular remodeling, and that such changes correlate with the severity of dyskinesia.

## Introduction

Parkinson’s disease (PD) is characterized by loss of dopaminergic neurotransmission in nigrostriatal pathways^1^. The clinical manifestations of the disorder can be treated with the dopamine precursor L-3,4-dihydroxyphenylalanine (L-DOPA), or levodopa^2^. During the past few years, it has become apparent that levodopa has pronounced localized effects on the neurovascular unit, a physiological entity comprised of neurons, astroglia, and juxtaposed endothelial cells^3^. Specifically, levodopa is transported across the blood-brain-barrier (BBB) by the large neutral amino acid transporter (LAT1), which is expressed on the endothelial cells. Upon crossing the BBB, the drug is regionally decarboxylated to dopamine, which is stored in presynaptic monoaminergic terminals^4^.

In addition to repleting striatal dopamine, levodopa administration corrects the elevations in local metabolic activity seen in PD subjects and 6-hydroxydopamine (6-OHDA) rodents studied in the baseline unmedicated state^5–7^. Indeed, a consistent relationship has been observed between the loss of nigral dopaminergic projections to the putamen and metabolic increases in the same brain region^8,9^. Interestingly, dual tracer imaging studies conducted in the two species revealed stereotyped dissociation of the metabolic (cerebral metabolic rate, CMR) and vasomotor (cerebral blood flow, CBF) responses to levodopa in dopaminoceptive brain regions^6,10^. We have found that levodopa-mediated dissociation effects localized to the putamen are a stereotyped feature of acute drug administration in human PD subjects^10^ and that these responses are exaggerated in patients with levodopa-induced dyskinesia (LID)^11^. This complication of chronic levodopa treatment, which involves the induction of abnormal, disturbing involuntary movements by the drug, affects approximately 50% of PD patients within five years of the initiation of therapy^12,13^. Indeed, dual tracer [^15^O]-labeled water ([^15^O]-H_2_O) and [^18^F]-fluorodeoxyglucose (FDG) PET data from human PD patients, demonstrated that acute levodopa administration causes local CBF in the putamen dissociation region to rise to abnormally high levels in association with these movements^11^. Moreover, LID patients exhibit marked uncoupling of cerebral blood flow and metabolism in this region during drug administration^11^.

How does the presence of levodopa-mediated uncoupling, with concomitant elevations in striatal on-state CBF, facilitate the appearance of LID? One possibility is that the intraluminal shear stress can increase as a consequence of a pronounced vasodilation, leading to localized BBB leakage in the medicated “on” state^10,14^. While less likely with normally formed vasculature, this possibility is more tenable in the setting of localized angiogenesis, given that immature blood vessels can form within dopaminergically denervated brain regions^15–18^.

In the current study, we investigated the relationship between the vasomotor and metabolic levodopa responses in 6-OHDA animals that developed drug-induced abnormal involuntary movements (AIMs), the rodent equivalent of LID, in the course of chronic daily treatment with this drug. To this end, we implemented a novel multitracer microPET technique to identify regions with significant CBF-CMR dissociation in response to levodopa injection, as well as local changes in BBB permeability, before and after levodopa administration. Thus, each animal was scanned with [^15^O]-H_2_O and [^18^F]-FDG, to map regional CBF and CMR, and with [^11^C]-isoaminobutyric acid (AIB) to assess BBB permeability. Scanning was conducted in a baseline state (PRE), following 6-OHDA lesioning but before initiation of daily levodopa treatment. The animals were then rescanned with all three tracers after 21 days of drug treatment, following the acute injection of saline (OFF) and levodopa (ON) in separate microPET sessions. During the treatment period, the animals were evaluated for the development and severity of levodopa-induced AIMs according to a well-established protocol^19^. The scan data were correlated with these ratings and with histopathological markers of angiogenesis in individual post-mortem brain samples.

## Results

### Dissociation of vasomotor and metabolic levodopa responses

**Figure 1:**
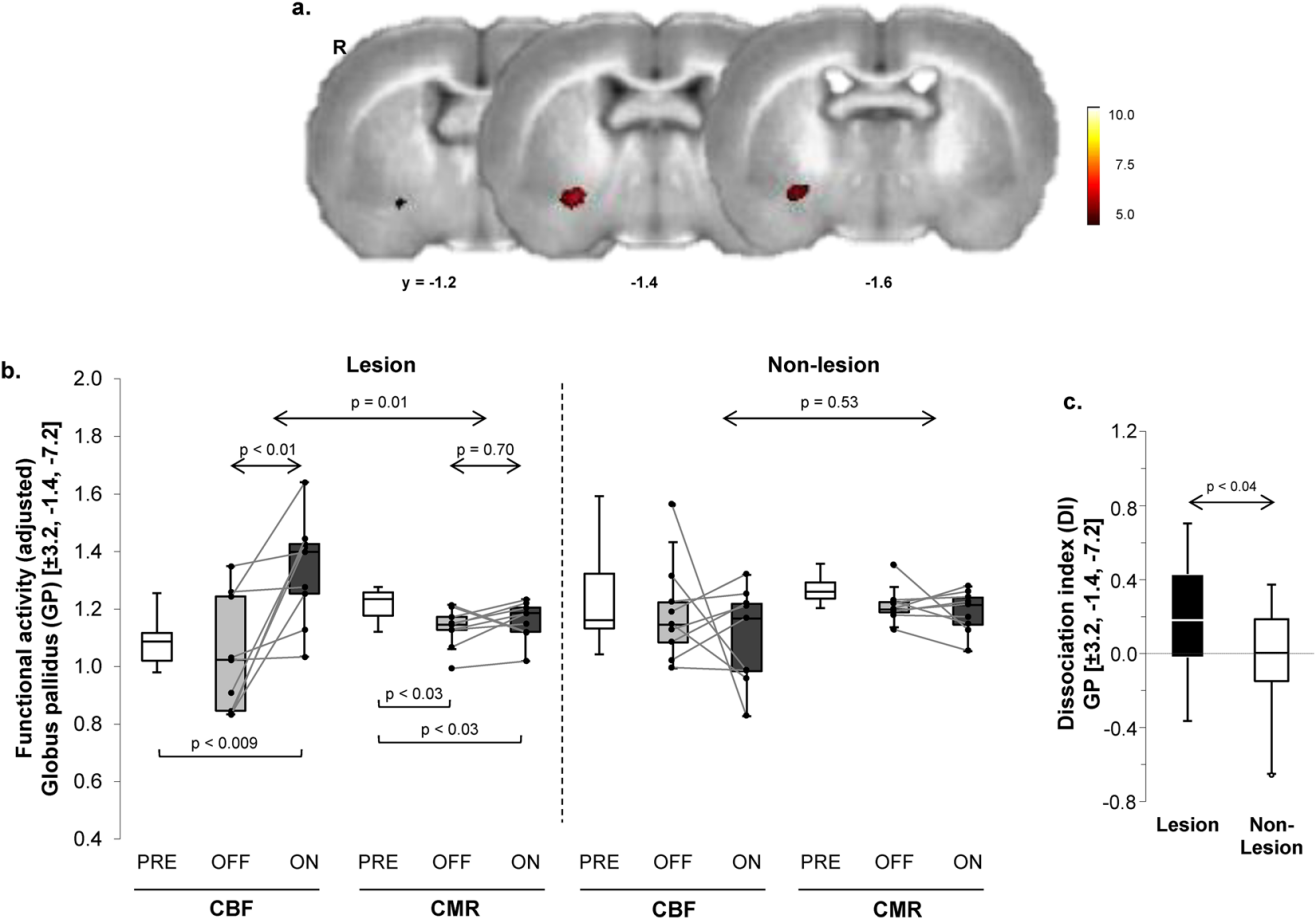
Dissociation of vasomotor and metabolic response to levodopa in the unilateral 6-OHDA rat dyskinesia model. (**a**) Voxel-wise searches over the whole brain volume revealed a distinct region in which local vasomotor (CBF) and metabolic (CMR) changes were significantly dissociated in response to levodopa. This cluster (*red*), comprised of 49 contiguous voxels (1 voxel = 0.8 × 0.8 × 0.8 mm) located at the border of the striatum and the ventral globus pallidus (GP) of the dopaminergically denervated right (R) cerebral hemisphere, was significant at a voxel-level threshold of p < 0.001 corrected for extent at p < 0.05 (see text). (**b**) Box-and-whisker plots of CBF and CMR values in this region. *Left*: Significant CBF–CMR dissociation was seen in the lesioned hemisphere (F_(1,8)_ = 10.85, p = 0.01, tracer × condition interaction effect; RMANOVA) with increased CBF (p < 0.01; post-hoc test) and no change in CMR (p = 0.70) in the OFF vs. ON condition. *Right*: Analogous changes were not seen in the contralateral non-lesioned hemisphere (p = 0.53). [Horizontal bars below box plots represent paired Student’s *t*-tests.] (**c**) Levodopa-mediated dissociation responses in this region were measured in the dopaminergically denervated and non-denervated hemispheres (see Methods). Dissociation responses were significantly greater on the 6-OHDA lesioned side relative to its non-lesionsed counterpart (p < 0.04; paired Student’s *t*-test).

To identify areas in which vasomotor and metabolic responses to single levodopa injections were significantly dissociated, we conducted voxel-wise searches over the entire brain volume for regions with significant tracer (CBF/CMR) × condition (ON/OFF) interaction effects (see Methods). The analysis revealed a significant levodopa-mediated dissociation region (Fig. 1a; Table 1), which was situated at the junction of globus pallidus (GP) and ventrocaudal striatum on the lesioned (right) cerebral hemisphere. Graphical inspection of individual CBF and CMR values for this region measured in the two conditions (Fig. 1b, *left*) disclosed a significant local interaction effect (F_(1,8)_ = 10.85; p = 0.01; 2 × 2 RMANOVA), with levodopa-mediated increases in normalized CBF (p < 0.01) and no corresponding change in CMR (p = 0.70; post-hoc tests). Analogous dissociation effects were not seen (F_(1,8)_ = 0.42, p = 0.53) in the contralateral “mirror” region on the non-lesioned (left) hemisphere (Fig. 1b, *right*). Indeed, the degree of levodopa-mediated dissociation in this region (Fig. 1c), represented in each hemisphere by the local dissociation index (DI = ΔCBF_ON–OFF_ – ΔCMR_ON–OFF_; see Methods) value, differed significantly for the two sides (p < 0.04; paired Student’s *t*-test). This indicates that the dissociation effect in this region was present only in the denervated hemisphere. Of note, no difference was observed (p > 0.57; Student’s *t*-tests) for GP CBF and CMR values measured in the pre-treatment (PRE) condition for the non-lesioned hemisphere and corresponding values measured in the sham-lesioned control group.

**Table 1:** Regions of Dissociated Vasomotor and Metabolic Levodopa Responses.

Voxel Search	Coordinates			Cluster Size (Voxels)	Z_max_	p-value^a^	CBF		CMR		Interaction Effect (VOI Analysis)	
	x	y	z				OFF/PRE	ON	OFF/PRE	ON	F-value^c^	p-value
CBF/CMR × OFF/ON	3.2	−1.4	−7.2	49	3.78	p < 0.001	1.04(0.20)^b^	1.34(0.18)	1.13(0.07)^**b**^	1.16(0.07)	10.85	0.011
CBF/CMR × PRE/ON	3.4	−1.4	−7.4	107	4.12	p < 0.001	1.05(0.10)	1.29(0.20)	1.21(0.05)	1.17(0.08)	16.42	0.005

^a^Corrected for cluster extent at p ≤ 0.05.

^b^Mean(SD) for local cerebral blood flow (CBF) and cerebral metabolic rate for glucose (CMR) in each of the significant clusters.

^c^F-tests for tracer × condition interaction effects in each volume-of-interest (VOI); repeated measured analysis of variance (RMANOVA).

PRE = drug-naïve baseline condition; OFF = saline injection; ON = levodopa injection following three weeks of daily drug administration (see text).

### Levodopa alters blood-brain-barrier transport

**Figure 2:**
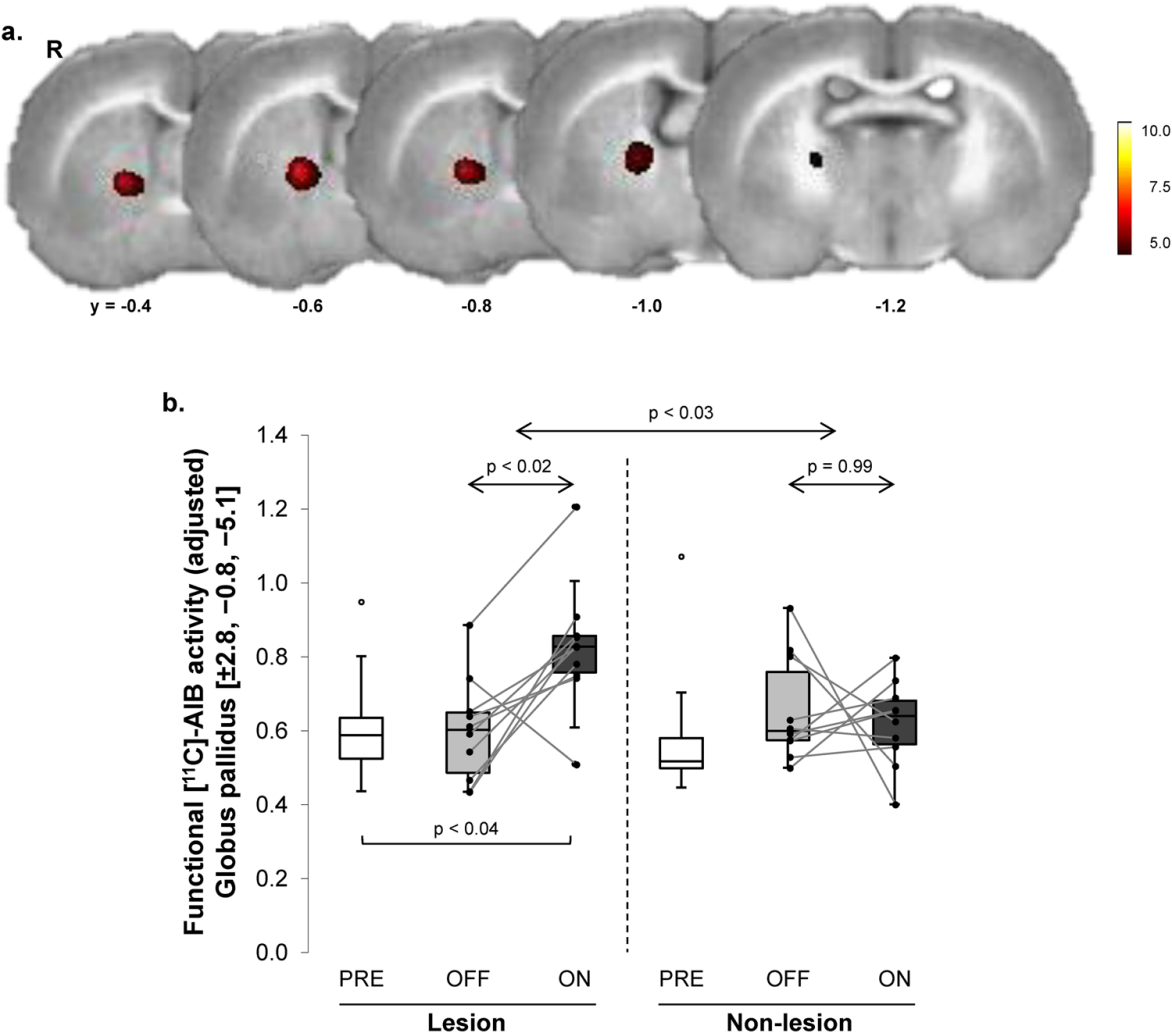
Levodopa-mediated changes in [^11^C]-AIB uptake in the unilateral 6-OHDA rat dyskinesia model. (**a**) Voxel-wise searches over the whole brain volume revealed a distinct area of increased [^11^C]-AIB uptake across the blood-brain-barrier (BBB) in responses to levodopa. This cluster (*red*) was comprised of 159 contiguous voxels (1 voxel = 0.8 × 0.8 × 0.8 mm) located in the globus pallidus (GP) of the dopaminergically denervated right (R) cerebral hemisphere. (**b**) Box-and-whisker plots of [^11^C]-AIB uptake values for this cluster measured in the PRE, OFF, and ON levodopa conditions (see text). There was a significant condition (ON/OFF) × hemisphere (lesioned/non-lesioned) interaction in this region (F_(1,9)_ = 7.92, p < 0.03; RMANOVA), with a significant effect on the denervated side (p < 0.02; post-hoc test). Analogous changes were not seen in the contralateral non-lesioned hemisphere (p = 0.99). [Horizontal bar below box plot represents paired Student’s *t*-test].

The effect of levodopa on local [^11^C]-AIB uptake, a measure of influx of the amino acid across the BBB, was evaluated in the same animals. To identify areas in which single levodopa injections increased local [^11^C]-AIB uptake, scans acquired in the ON and OFF conditions were interrogated voxel-wise over the entire brain volume. A significant increase in [^11^C]-AIB uptake (Fig. 2a; Table 2) was noted in the GP of the lesioned hemisphere. A significant hemisphere × condition interaction effect (Fig. 2b) was present in this region (F_(1,9)_ = 7.92, p < 0.03; 2 × 2 RMANOVA), with levodopa-mediated increases in [^11^C]-AIB uptake on the lesioned side (p < 0.02) but not on the contralateral non-lesioned side (p = 0.99; post-hoc tests). [^11^C]-AIB uptake values in this region measured in the PRE condition did not differ significantly for the non-lesioned hemispheres of the 6-OHDA animals vs. their sham-lesioned control counterparts (p = 0.42; Student’s *t*-test; data not shown).

**Table 2:** Regions of Increased [^11^C]-AIB Uptake.

Voxel Search	Coordinates			Cluster Size (Voxels)	Z_max_	p-value^a^	[^11^C]-AIB Uptake		Levodopa Effect (VOI Analysis)	
	x	y	z				OFF/PRE	ON	T-value^c^	p-value
ON > OFF	2.8	−0.8	−5.1	159	4.29	p < 0.001	0.60(0.14)^**b**^	0.83(0.17)	3.59	0.006
ON > PRE	2.6	−0.7	−4.9	144	4.20	p < 0.001	0.76(0.16)	0.95(0.13)	3.40	0.009

^a^Corrected for cluster extent at p ≤ 0.05.

^b^Mean(SD) for local [^11^C]-AIB uptake in each of the significant clusters.

^c^T-values for changes in each volume-of-interest (VOI); paired Student’s t-tests.

PRE = drug-naïve baseline condition; OFF = saline injection; ON = levodopa injection following three weeks of daily drug administration (see text).

### Effects of daily levodopa administration prior to imaging

We next determined whether the acute effects of levodopa administration, as observed in the ON vs. OFF contrasts, were influenced by changes in the baseline unmedicated condition that resulted from the preceding three-week period of daily drug administration. To this end, we compared the OFF scans for each tracer with the corresponding PRE scans, which were acquired in the pre-treatment baseline condition. Significant regional differences were not seen on voxel-wise comparisons of hemispheric maps of CBF, CMR, and [^11^C]-AIB uptake acquired in the PRE and OFF conditions. We additionally conducted post-hoc comparisons of OFF values for the three tracers measured in the significant clusters identified above. In the levodopa dissociation region (depicted in Fig. 1a), significant declines in local CMR (Fig. 1b) were noted in OFF relative to PRE (p < 0.03; paired Student’s *t*-test). Analogous baseline changes were not present for CBF and [^11^C]-AIB uptake measurements in this region (p > 0.19). Indeed, in this region, levodopa-mediated changes in ON relative to PRE were significant for each of the three tracers (CMR: p < 0.03; CBF: p < 0.008; [^11^C]-AIB: p < 0.04; paired Student’s *t*-tests).

The levodopa-mediated dissociation effect seen in the striatum/ventral GP of the lesioned side was substantiated by a separate voxel-wise search for significant tracer × condition interactions involving the ON and PRE scans. Indeed, this analysis revealed a significant dissociation cluster in the GP of the lesioned hemisphere (Table 1), which was in close proximity to the original interaction region. Similarly, in the comparison of [^11^C]-AIB scans acquired in the ON vs. PRE conditions, we noted an area of increased uptake (Table 2), which was also in close proximity to that reported above for the ON vs. OFF contrast.

### Correlations of the imaging measures with levodopa-induced dyskinetic movements

**Figure 3:**
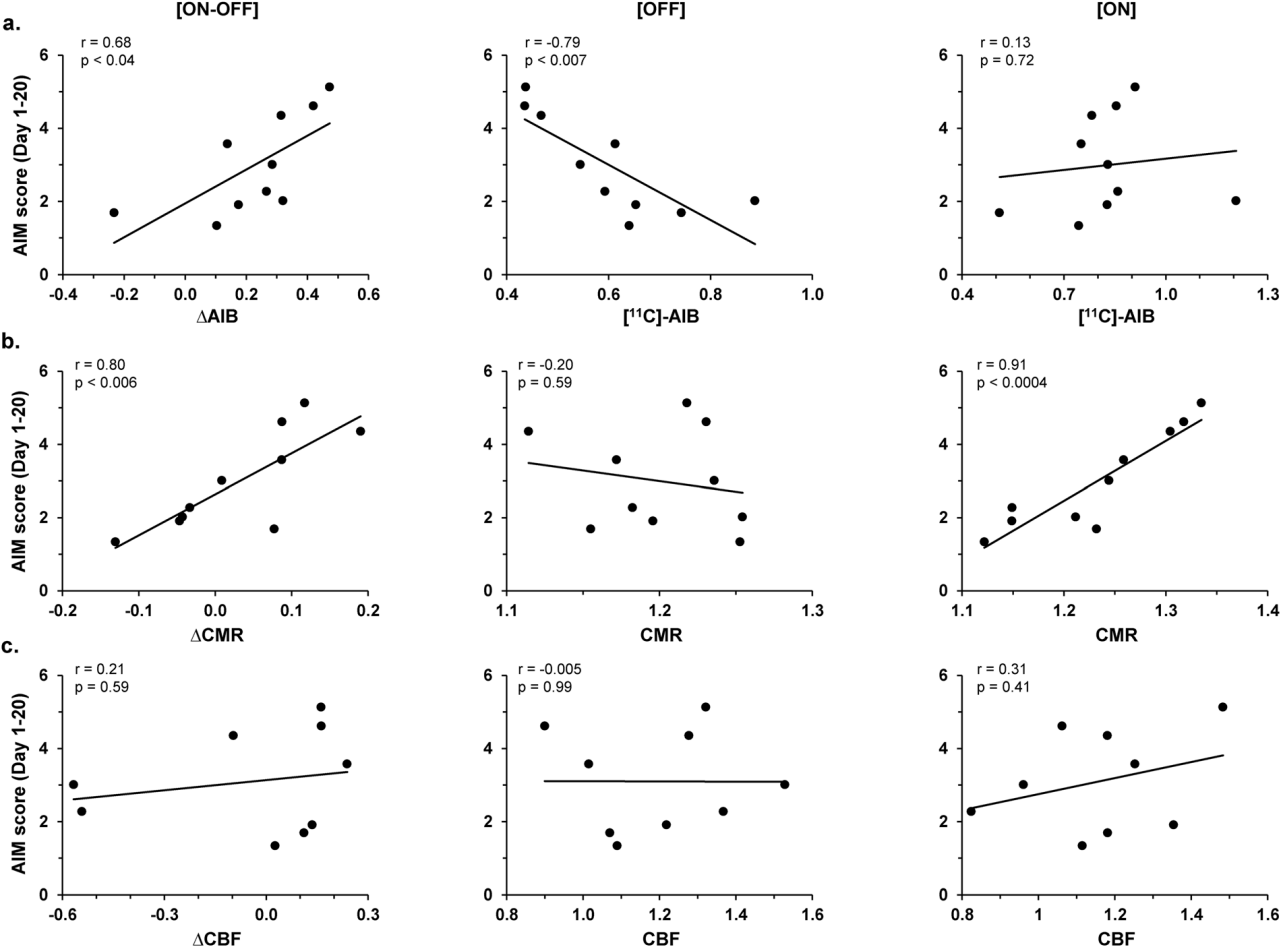
Correlations with abnormal involuntary movements. (**a**) Composite AIMs scores averaged over days 1–20 of levodopa administration significantly correlated (r = 0.68, p < 0.04) with levodopa-mediated changes in [^11^C]-AIB uptake (∆AIB, *left*) measured in the significant GP cluster on the lesioned hemisphere (Fig. 2a). The composite AIMs scores correlated with [^11^C]-AIB values measured in the OFF (r = −0.79, p < 0.007; *middle*) but not in the ON (r = 0.13, p = 0.72; *right*) conditions. (**b**) Analogous correlation of levodopa-mediated changes in local metabolic activity (∆CMR, *left*) measured with corresponding AIMs scores was also significant (r = 0.80, p < 0.006). Nonetheless, AIMs correlations with CMR values in this region were significant for the ON condition (r = 0.91, p < 0.0004; *right*) but not for OFF (r = −0.20, p = 0.59; *middle*). (**c**) By contrast, the composite AIMs scores did not correlate (p > 0.41) with levodopa-mediated changes in cerebral blood flow (∆CBF, *left*) or CBF values in the OFF (*middle*) or ON (*right*) condition measured in this region.

To determine the relationship of the imaging measures to levodopa-induced abnormal movements, we examined the correlations between the composite AIMs scores for the individual animals and the corresponding ON-OFF vasomotor or metabolic changes (∆CBF and ∆CMR) recorded in the striatal/ventral GP dissociation region (Fig. 1a) of the lesioned hemisphere. Likewise, composite AIMs scores were correlated with levodopa-mediated changes in [^11^C]-AIB uptake (∆AIB) recorded in the ipsilateral GP region (Fig. 2a) of the same animals. Correlations with AIMs scores were not significant for tracer changes in the levodopa dissociation region (p > 0.78; Pearson’s correlations). Nonetheless, AIMs scores in the same animals correlated with ∆AIB values measured in the GP area of increased tracer uptake. Composite AIMs scores (Fig. 3a,b, *left*) correlated with ∆AIB (r = 0.68, p < 0.04; Pearson’s correlation) and with ∆CMR (r = 0.80, p < 0.006) values measured in this region of the lesioned hemisphere. The correlation with local ∆CBF values (Fig. 3c, *left*), however, was not significant (p = 0.59). Of note, ∆AIB and ∆CMR values measured in this region were not significantly related (R^2^ = 0.04, p = 0.59; linear regression). Thus, changes in GP uptake for the two tracers served as independent predictors of the composite AIMs scores in these animals, together accounting for over 90% of individual differences in outcome (model R^2^ = 0.92, p = 0.0001; multiple linear regression). By contrast, composite AIMs scores did not correlate (r < 0.44, p > 0.20) with ∆AIB, ∆CMR, or ∆CBF values measured on the contralateral non-lesioned side.

### Upregulation of angiogenesis markers

**Figure 4:**
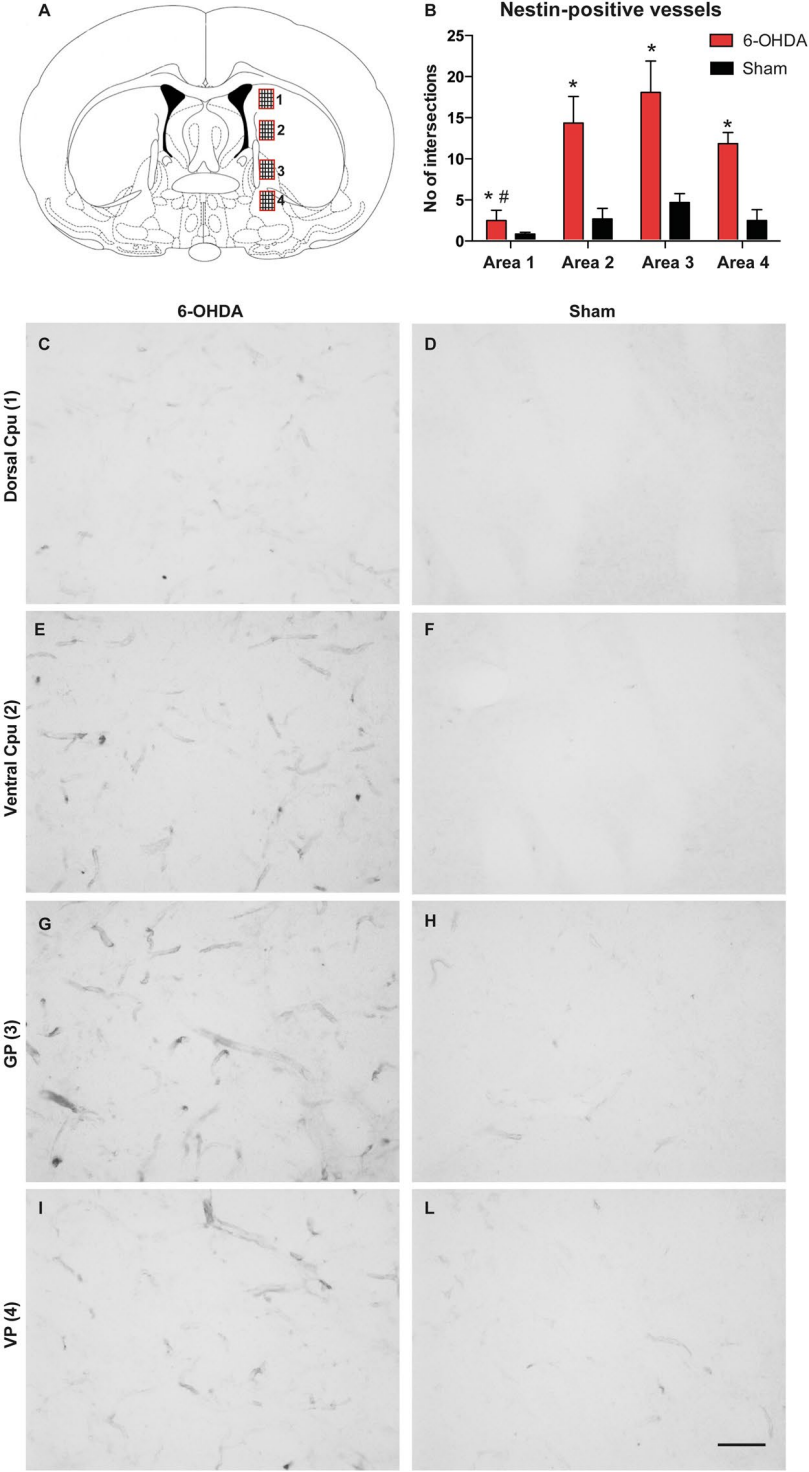
Nestin is significantly upregulated in the globus pallidus of dyskinetic rats. (**A**) The density of nestin-immunoreactive vessels was estimated by counting the number of intersections formed by immunopositive vessels on a grid that fully covered each sample areas (see Methods). [Four sample areas (1–4) were digitized from each animal spanning the dorsoventral extent of the caudate-putamen and the pallidum.] (**B**) A measure of nestin-immunoreactive microvessels (number of intersections) indicates largest density of stained microvessels in the GP (Area 3), and lowest density in the dorsal CPu (Area 1) (see text). [Post-hoc Bonferroni test, *p < 0.05 vs. Sham, # vs. Ventral CPu and GP).] (**C**–**L**) Representative photomicrographs were taken under a 20 × objective to show nestin-immunoreactive microvessels in the dorsal Cpu (**C**,**D**), Ventral Cpu (**E**,**F**), GP (**G**,**H**) and VP (**I,L**) from 6-OHDA-lesioned and sham-lesioned controls, respectively. [Scale bar, 50 µm. Cpu = caudate-putamen; GP = globus pallidus; VP = ventral pallidum.]

After the animals were sacrificed, sections through the striatum and GP were immunostained for nestin, which is upregulated in immature endothelial cells in brain regions undergoing angiogenesis^15,20,21^. Microvessel nestin immunoreactivity was quantified through the dorsoventral extent of the striatum and pallidum (Fig. 4A). Sample areas were digitized from levels that included the node of [^11^C]-AIB leakage seen on the microPET scans. This quantitative analysis revealed highly significant overall differences between groups and sample areas (Group: F_(1,40)_ = 38.89; p < 0.0001; Area: F_(3,40)_ = 7.9; p = 0.0003; Interaction: F_(3,40)_ = 3.2; p = 0.0332).

Compared to sham-lesioned controls (also treated with levodopa), the 6-OHDA lesioned dyskinetic animals showed significant upregulation of nestin-immunostained microvessels in each region (Fig. 4B). Within the dyskinetic group, nestin immunoreactive microvessels were less frequent in the dorsal caudate-putamen (Fig. 4C), and most prominent in the GP (Fig. 4G).

## Discussion

Previous PET imaging studies in PD patients have linked LID to localized flow-metabolism dissociation in putaminal and adjacent pallidal regions^10,11^. By applying a multimodal microPET imaging approach to a rat model of LID, we now show that levodopa-mediated flow-metabolism dissociation is accompanied by a localized increase in BBB permeability, which correlated with the composite AIMs ratings of LID severity.

The finding of levodopa-induced flow–metabolism dissociation in the striatum/GP of dyskinetic rats is in keeping with similar results obtained in chronically medicated PD patients affected by LID^10,11^, as well as with the increases in local dopamine release in response to levodopa seen in these individuals^22^. Here, however, the same animals additionally underwent concurrent [^11^C]-AIB microPET to assess levodopa-mediated changes in regional BBB function. We found significant increases in the uptake of this radiotracer in the GP of the lesioned hemisphere, which correlated with the severity of drug-induced abnormal movements observed in the individual animals. Indeed, the association of focal [^11^C]-AIB leakage with nestin-positive microvessels in the involved regions supports the hypothesis that the increase in BBB permeability associated with LID is intimately linked with angiogenesis^4^.

The finding of dissociated vasomotor and metabolic levodopa responses in the caudate-putamen and GP on the lesioned hemisphere is consistent with an earlier autoradiographic study conducted in the same rat model^16^. In that study, chronically levodopa-treated animals with drug-induced abnormal movements exhibited higher on-state CBF in the striatum and GP than animals without abnormal movements; analogous differences in CMR were not discerned under the same treatment conditions^16^. Although microPET has lower anatomical resolution than autoradiography, *in vivo* imaging allowed for CBF and CMR to be measured concurrently in the same animals scanned on and off drug^6^. Moreover, this approach allowed us to conduct a voxel-wise search of the whole brain to localize regions with significant dissociation effects without the use of pre-specified regions-of-interest (ROIs).

In human PD, the levodopa-mediated flow-metabolism dissociation is associated with abnormal baseline elevations in glucose metabolism in the putamen and GP, which decline consistently with drug^10,11^. While significant increases in baseline metabolic activity have also been described in homologous brain regions of the non-human primates with MPTP (1-methyl-4-phenyl-1,2,3,6-tetrahydropyridine) parkinsonism^23–25^, evidence has been less consistent for corresponding metabolic changes in the rat model. In the autoradiographic study of Ohlin *et al*.^16^, dopaminergic denervation was associated with increased 2-DG (2-deoxy-D-glucose) uptake in the rat GP at baseline that declined following chronic levodopa administration. While baseline metabolic increases were not observed with microPET, a significant decline was detected following three weeks of daily drug in both the medicated (ON) and unmedicated (OFF) conditions relative to the pre-treatment baseline (PRE). It is unknown whether the observed differences between species are attributable to biological effects, technical factors, or both.

Also in general agreement with the earlier autoradiographic study^16^, we found evidence of levodopa-mediated increases in [^11^C]-AIB uptake in the basal ganglia of the lesioned hemisphere in chronically treated 6-OHDA rats. It is also noteworthy that [^11^C]-AIB uptake in this region was negligibly low in both hemispheres when measured before the initiation of daily levodopa treatment (PRE) or in the off-state (OFF) after receiving the drug daily for three weeks. Thus, the localized changes in [^11^C]-AIB uptake observed in this study was clearly induced by levodopa administration. We have suggested that the on-state CBF-CMR dissociation may alter BBB permeability^11^. Indeed, studies in PD patients indicate that vasomotor effects of levodopa can cause local CBF to rise to abnormal levels. It is however doubtful that the observed increases in regional perfusion are sufficient in themselves to disrupt the integrity of the vascular endothelium^11^. Nevertheless, an increase in local blood flow can trigger BBB leakage through immature, angiogenic vessels. Angiogenesis has been found to occur in chronically levodopa-treated 6-OHDA rats^14,15,17^ and in all likelihood also in PD patients with LID^16,26^. Interestingly, in the current set of dyskinetic animals, the region exhibiting largest density of nestin-immunoreactive microvessels was the GP, closely matching the area of [^11^C]-AIB leakage seen on microPET. That said, nestin-immunoreactive microvessels were additionally seen in other striatal and pallidal regions. The comparatively low spatial resolution of rodent [^11^C]-AIB microPET imaging may require a substantial degree of ongoing angiogenesis for a significant locus of BBB leakage to be discerned. Moreover, with resolutions of 1.83 and 1.49 mm for O-15 and C-11, respectively, it may be difficult to identify discrete areas of increased flow-metabolism dissociation and altered BBB permeability within small subregions of the rodent basal ganglia.

We note that levodopa may also affect regional [^11^C]-AIB transport across the BBB through mechanisms unrelated to its local vasomotor effects. Indeed, pharmacologic activation of dopamine receptors has recently been found to interfere with gap junction communication in local neurons and astrocytes^27^, which may alter BBB permeability on a regional basis. Irrespective of cause, the observed levodopa-mediated increase in pallidal [^11^C]-AIB uptake is biologically relevant in that these changes correlated with the severity of the drug-induced involuntary movements. Of note, the [^11^C]-AIB changes in the GP were also unrelated to corresponding changes in local glucose utilization, an index of synaptic activation in the region. That said, both ∆AIB and ∆CMR measured in the GP cluster correlated with independent AIMs scores, each explaining different aspects of LID severity. While validation of these relationships is needed, one can attribute each of the effects to discrete mechanisms: [^11^C]-AIB uptake to input, in that it measures local BBB permeability, which may be under dopaminergic control^27,28^, and CMR to output, in that it measures synaptic activity, which may be regulated by dopamine receptors on GP neurons^29^.

The relationship of the localized BBB changes seen with chronic levodopa exposure to ongoing regional angiogenesis is intriguing. Indeed, angiogenesis and BBB formation are intimately linked processes in normal brain development. Wnt/β-catenin signaling induces not only central nervous system angiogenesis but also expression of BBB components GLUT1 and Claudin-3^30–33^. Indeed, pathways involved in BBB formation may be affected by both genetic and environmental factors associated with PD^34–37^. Lastly, the current data link angiogenesis to regions with altered BBB transport. Indeed, the current findings accord with prior histopathological data from dyskinetic 6-OHDA rats^14,17^ and human PD patients with LID^16,26^, showing upregulation of angiogenesis markers in the involved striatum and GP regions. The altered BBB properties associated with angiogenesis may contribute to uncontrolled and uneven levodopa delivery, as well as glial activation and neuroinflammation, which favor the development of LID^38,39^.

## Methods

### Animals and procedures

#### Animals

Female Sprague-Dawley rats (200−350 g; Harlan, The Netherlands) were housed under a 12 h light/dark cycle with free access to food and water. All procedures were in accordance with National Institutes of Health guidelines and approved by the Malmo-Lund Ethical Committee on Animal Research (Lund, Sweden) and by the Institutional Animal Care and Use Committee (IACUC) at The Feinstein Institute for Medical Research (Manhasset, NY).

#### Drugs

L-DOPA methyl ester and benserazide hydrochloride (peripheral DOPA decarboxylase inhibitor) (Sigma-Aldrich, Stockholm, Sweden) were freshly dissolved in saline and co-administered subcutaneously (s.c.) at the doses of 6 and 12 mg/kg, respectively. The injection volume was 1.0 ml/kg body weight.

#### Dopamine-denervating lesioning and behavioral screening

Ten rats received a unilateral injection of 6-OHDA hydrochloride (Sigma-Aldrich) into the right ascending dopamine fiber bundle (medial forebrain bundle), as previously described^6,16^. Thirteen sham-lesioned animals received injections of vehicle at the same coordinates. Two weeks following surgery, rats were tested for amphetamine induced-rotation (2.5 mg/kg d-amphetamine intraperitoneally (i.p.), 90 min recordings). Only animals exhibiting >5 net full turns per minute in the direction ipsilateral to the lesion were selected for the experiments^40^. The 6-OHDA lesion was verified with tyrosine hydroxylase immunohistochemistry in all animals at the conclusion of the study.

#### Experimental design

Multimodal PET data were acquired from rats with unilateral 6-OHDA lesions of the medial forebrain bundle (n = 10) or sham lesions (n = 13). Rats received a single s.c. injection of saline 30 minutes before anesthesia induction with isofluorane and scanned before treatment on a Siemens Inveon (Siemens, Munich, Germany) at The Feinstein Institute for Medical Research. Following completion of a pre-treatment scan (PRE), 6-OHDA lesioned rats received two daily s.c. injections of levodopa (6 mg/kg) plus benserazide (12 mg/kg) for six days per week for a total of three weeks (21 days), which served as chronic levodopa treatment. AIMs were assessed according to well-validated criteria (See *Behavioral assessment* section below).

Upon completion of chronic treatment, animals underwent scanning under two conditions: after s.c. injections of saline (OFF) or levodopa (ON). OFF and ON scans occurred one week apart; half of the animals underwent OFF scanning first, and the other half underwent ON scanning first. During the week between the OFF and ON scanning sessions, after completion of their 21-day treatment and until the time of their transcardial perfusion, animals continued receiving one daily s.c. levodopa injection, for treatment and dyskinesia maintenance.

#### MicroPET

Rats were injected with s.c. saline or levodopa 30 minutes prior to scan acquisition and 15 minutes before anesthesia induction with 3.5−4% isofluorane in 100% oxygen via a breathing mask. Once absence of reflexes was confirmed, the lateral tail vein was catheterized for [^15^O]-H_2_O injection and a 25-gauge butterfly catheter line was secured with Transpore tape (3 M, Maplewood, MN) in the right intraperitoneal cavity for [^18^F]-FDG injection, as previously described^6^. After the lines were secured, the animal was placed on the scanner platform, and anesthesia delivery was reduced to 1.5−2% in 100% oxygen for the remainder of the scan. The animal’s position was maintained until completion of the scanning session. To measure CBF, 1−2 mCi of [^15^O]-H_2_O tracer was injected into the lateral tail vein line and a 4-minute dynamic emission scan was immediately begun. One animal with an anomalous CBF value in the OFF condition was excluded from the dissociation analysis.

After allowing 10 minutes for radiotracer decay, we injected 1−2 mCi of [^11^C]-aminoisobutyric acid (AIB) to assess the integrity of the BBB. [^11^C]-AIB is an inert, neutral amino acid that does not readily cross the BBB (under normal conditions. Its transport across the BBB is mediated through Na^+^-dependent system A, and is normally inefficient^41^). Following intravenous injection, [^11^C]-AIB is concentrated by capillary endothelial cells, and moves bidirectionally from blood to brain and back. Regional [^11^C]-AIB uptake can increase, however, under pathological conditions involving change in BBB permeability, amino acid transport, or both.

In this study, [^11^C]-AIB was injected into the tail vein catheter and data were acquired during a 90-minute dynamic emission scan and a subsequent 10-minute transmission scan. To maintain a consistent ON (or PRE/OFF) state during [^11^C]-AIB imaging, we gave the animals a s.c. injection of levodopa (or saline) one hour after radiotracer injection. After completion of the [^11^C]-AIB scan, 1−2 mCi of [^18^F]-FDG was injected into the previously secured right i.p. line and a 45-minute uptake period was allowed prior to the acquisition of a 10-minute emission scan and a 4-minute transmission scan. At the end of the scanning session, animals were allowed to recover in a clean cage until they regained their righting reflex.

#### Behavioral assessment

During the treatment period, we rated the AIMs of the animals three to four times a week after the morning levodopa injection, according to well-validated criteria^19^. Briefly, we observed each rat for AIMs for 1 minute every 20 minutes following drug injection, for a total duration of 180 minutes. Axial AIMs (dystonic posturing or twisting movements of the neck and upper body towards the side contralateral to the lesion), limb AIMs (purposeless movements of the contralateral forelimb), and orolingual AIMs (empty jaw movements and contralateral tongue protrusion) were scored on a severity scale from 0 to 4, based on the proportion of observation time during which the dyskinesia was present. A total AIMs score for each animal was obtained by summing the severity score for each dyskinesia subtype from each monitoring period. Each total score was divided by nine to obtain a numerical score for each observation period (a total of 9 one-minute observation periods). The composite behavior was defined as the average of scores from every rated treatment day (days 1−20).

### Image analysis

#### Data processing

Imaging data was processed using PMOD (PMOD Technologies LLC, Zurich, Switzerland) and SPM5 (Wellcome Trust Centre for Neuroimaging, London, UK) with SPMMouse (Wolfson Brain Imaging Centre, University of Cambridge, Cambridge, UK; www.spmmouse.org) implemented in Matlab 6.1 (MathWorks, Natick, MA). [^18^F]-FDG scans from each animal were manually aligned to an [^18^F]-FDG template in PMOD^42^. The transformations from the [^18^F]-FDG scan to the template were then applied to the corresponding [^15^O]-H_2_O and [^11^C]-AIB scans in each animal. (Regarding the [^11^C]-AIB scan, the final three frames (60–90 minutes post injection) of each decay-corrected scan were averaged and used for analysis.) The scans of all the animals were subsequently aligned to each other and were registered together to a common MRI template in SPM5. Final alignments were visually inspected using PMOD software. Images were smoothed with an isotropic Gaussian kernel FWHM (full width at half maximum) 0.8 mm at all directions to improve the signal-to-noise ratio.

#### [^15^*O*] − *H*_*2*_*O and* [^18^*F*] − *FDG*

To identify brain regions in which there was significant dissociation between CBF and CMR during levodopa administration, we conducted unbiased whole brain voxel-wise searches in the 6-OHDA-lesioned animals. This analysis identified regions from the [^15^O]-H_2_O and [^18^F]-FDG PET data where there were interaction effects between the tracers and conditions (PRE, ON [levodopa], and OFF [saline]) in the globally normalized scan data. Searches were performed for both PRE vs. ON and OFF vs. ON conditions. Areas with interaction effects were considered significant at a voxel-level threshold of p < 0.001, with a correction for cluster extent at p < 0.05.

#### [^11^*C*] − *AIB*

To identify regions with significant levodopa-mediated increases in [^11^C]-AIB uptake, we performed separate whole brain voxel-wise searches as described above for the other tracers. Specifically, we interrogated the data for clusters in which radiotracer uptake measured in decay-corrected scans acquired 60–90 minutes post injection was greater in the ON vs. OFF and in the ON vs. PRE conditions. As with the other tracers, levodopa-mediated changes in regional [^11^C]-AIB uptake were considered significant at a voxel-level threshold of p < 0.001, with a correction for cluster extent at p < 0.05.

#### Regional analysis

Brain regions identified through the above-mentioned whole-brain searches were analyzed with post-hoc volume-of-interest (VOI) analyses in order to evaluate individual data from each significant cluster. For each VOI, functional activity values for [^15^O]-H_2_O, [^18^F]-FDG, and [^11^C]-AIB were ratio-normalized by the corresponding global whole-brain value for each scan. For each significant VOI, globally normalized [^15^O]-H_2_O, [^18^F]-FDG, and [^11^C]-AIB values were measured for individual animal in each treatment condition (PRE, OFF, and ON). Additionally, we computed values for each tracer measured in the PRE, OFF, and ON conditions at “mirror” VOIs defined by reflecting the center of the cluster over the y-axis (from [x, y, z] to [−x, y, x]) as an internal control. Interaction effects in the CBF/CMR VOI data were evaluated by two-way 2 × 2 repeated measures ANOVA (RMANOVA), with ON/OFF or ON/PRE conditions and [^15^O]-H_2_O/[^18^F]-FDG scans as two within-subject variables. BBB effects were analyzed by two-way 2 × 2 RMANOVA with ON/OFF conditions as within-subject variable and lesion/non-lesion hemispheres as between-subject variable. Appropriate Bonferroni post-hoc corrections were applied for all analyses.

For the region of significant interaction effects, we also computed a regional dissociation index (DI), defined as the change in blood flow (ΔCBF_ON-OFF_ = CBF_ON_ − CBF_OFF_) minus the change in metabolism (ΔCMR_ON-OFF_ = CMR_ON_ − CMR_OFF_) for an individual animal^6,10,11^. In this computation, a DI value of 0 indicated equal treatment-mediated changes in functional activity for CBF and CMR scan data. Positive DI values indicated greater treatment-mediated changes in blood flow relative to metabolism (ΔCBF_ON–OFF_ > ΔCMR_ON–OFF_), whereas negative values indicated the opposite (ΔCBF_ON–OFF_ < ΔCMR_ON–OFF_). Differences in DI across the lesioned and non-lesioned hemispheres were assessed with paired Student’s *t*-test.

#### Behavioral correlations

To understand the relationship of dyskinesia with local CBF, CMR, and [^11^C]-AIB uptake, we performed correlation analyses of each animal’s axial, limb, and orolingual AIMs scores using the average composite score per test session from the entire treatment period (days 1−20). Individual differences in composite AIMs scores were correlated with levodopa-mediated changes (ON – OFF) in radiotracer activity computed for each VOI, and with local PRE, OFF, and ON values for each of the tracers. Correlations were considered significant for p ≤ 0.05 (Pearson’s correlation).

#### Histopathological analysis

After the completion of all scans, rats received 100 mg/ml of ketamine and 20 mg/ml of xylazine at a volume of 0.15 ml per 0.1 kg body weight before being transcardially perfused with 50 ml of 0.9% NaCl and 250 ml ice-cold 4% paraformaldehyde (PFA) in 0.1 M phosphate buffer. Brains were post-fixed in cold PFA for 2 hours, and subsequently transferred to a sucrose solution containing 0.05% PFA. Samples were refrigerated in this solution and shipped to Lund, Sweden for processing.

Free-floating immunohistochemistry was performed as previously described^14,17^. Endogenous peroxidases were quenched using 3% hydrogen peroxide and 10% methanol for 20 minutes. Sections were incubated in 0.02 M potassium-PBS with 0.1% Triton-X (KPBS/T) and blocked in a solution of 5% normal serum. The mouse anti-nestin primary antibody (concentration 1:8,000) (BD Biosciences, Franklin Lakes, NJ), used as a marker for immature endothelium, was incubated overnight at 4 °C in KPBS/T containing 5% normal serum. The secondary antibody, biotinylated horse anti-mouse antibody 1:200 (Vector Laboratories, Burlingame, CA), was incubated 1 hour at room temperature in KPBS/T with 2.5% normal serum. Antibody complexes were detected using a peroxidase-based method (with 3′3′-diaminobenzidine as the chromogen). Slide-mounted sections were cover-slipped with DPX mounting medium or polyvinyl alcohol-1,4-[2.2.2]-octane (Sigma-Aldrich). Nestin is expressed not only on blood vessels, where it is associated with angiogenesis^20^, but also by neuroepithelium-derived progenitor cells^43^. We therefore took special care to assess nestin staining only on blood vessel profile^17^.

Quantification of nestin-positive microvessels was performed by an experimentally blinded investigator on two sections per animal encompassing the regions of flow-metabolism dissociation and increased [^11^C]-AIB uptake (rostrocaudal levels + 0.02 mm and −0.10 mm from bregma in the Paxinos and Watson rat brain atlas^44^). The analysis was performed using both 6-OHDA-lesioned animals and sham-lesioned controls showing good tissue quality (n = 6 per group). Sample areas of equal size (353287 µm^2^/area) were digitized across the dorsoventral extent of caudate-putamen (CPu), globus pallidus (GP) and ventral pallidum (VP) on the side ipsilateral to the lesion (image acquisition on a Nikon 80i microscope under a 10 × objective). Nestin immunoreactivity on microvessels was quantified by overlaying a grid (2174 µm^2^/square) on each digitized area and counting the number of intersections formed by immunopositive vessels on the grid.

#### Statistical analysis

Statistical analysis was performed using Statistical Analysis System (SAS), version 9.3 (SAS Institute Inc., Cary, NC). The results were considered significant at p < 0.05.

#### Data availability

The datasets that were generated in the course of the current study can be made available on reasonable request from interested investigators.
